# Determination of frequency of spontaneous resistance for gepotidacin and levofloxacin against a collection of gram-positive and gram-negative organisms

**DOI:** 10.1128/aac.01832-25

**Published:** 2026-04-30

**Authors:** Gina M. Morgan, John H. Kimbrough, Maura H. Karr, Jennifer Thompson, Josh West, Mariana Castanheira, S. J. Ryan Arends, Nicole Scangarella-Oman

**Affiliations:** 1 JMI Laboratories/Element Materials Technology 138461, North Liberty, Iowa, USA; 2 GSK 525885 , Collegeville, Pennsylvania, USA; Shionogi Inc 71777 , Florham Park, New Jersey, USA

**Keywords:** gepotidacin, frequency of resistance, gepotidacin target-based mutations, SNP, resistance mechanisms

## Abstract

Gepotidacin is a novel, bactericidal, first-in-class triazaacenaphthylene antimicrobial recently approved by the Food and Drug Administration (FDA) and Medicines and Healthcare products Regulatory Agency for the treatment of uncomplicated urinary tract infections (uUTI) and by the FDA for uncomplicated urogenital gonorrhea. Gepotidacin selectively inhibits type II topoisomerases involved in bacterial DNA replication by a distinct binding site and novel mechanism of action, conferring *in vitro* activity against most uropathogens, including those resistant to current antimicrobials. This study evaluated the potential of gepotidacin to select for spontaneous resistance in *Citrobacter freundii*, *Enterobacter cloacae* species complex, *Klebsiella aerogenes*, *Klebsiella pneumoniae*, *Proteus mirabilis*, *Providencia rettgeri*, *Enterococcus faecalis*, and *Staphylococcus saprophyticus* clinical isolates. Mutant frequencies ranged from 6.1 × 10^−8^ to <2.5 × 10^−10^ at 4× gepotidacin MIC concentrations, and no mutant isolates were recovered at 10× gepotidacin MIC concentrations. No target gene-specific (*gyrA*, *gyrB*, *parC*, *parE*) cross-resistance was observed with any of the comparators tested. As gepotidacin target site mutations in *gyrA/B* were only found in *Enterococcus faecalis*, repressor inactivation or activator dysregulation mutations in efflux pumps were identified as possible mechanisms of reduced susceptibility in the Gram-negative isolates. Further investigation is needed into the relevance of these resistance mechanisms, the clinical significance of these findings, and whether there are any potential fitness costs of decreased gepotidacin susceptibility.

## INTRODUCTION

Infections caused by antimicrobial-resistant organisms account for more than 35,000 deaths per year in the United States and were directly responsible for 1.27 million deaths worldwide in 2019 alone (1, 2). Persistent use of broad-spectrum antimicrobials has selected for cross-resistance due to structural similarities and redundant modes of action, limiting antimicrobial therapy choices and contributing to the rise of multidrug-resistant (MDR) bacteria (3–5). Of special concern are the opportunistic ESKAPEE organisms group (*Enterococcus faecium*, *Staphylococcus aureus*, *Klebsiella pneumoniae*, *Acinetobacter baumannii*, *Pseudomonas aeruginosa*, *Enterobacter* spp., and *Escherichia coli*), which are pathogens with high rates of MDR, increasing virulence, and are the leading cause of nosocomial infections worldwide (6, 7). Efficient at producing genetic mutations and exchanging genetic material via mobile genetic elements, the ESKAPEE pathogens have developed drug resistance against a wide variety of antimicrobial classes, including carbapenems, fluoroquinolones, macrolides, and tetracyclines (6, 8–11). The Gram-negative members of the ESKAPEE group are notorious extended-spectrum β-lactamase (ESBL) producers not only resistant to most β-lactam agents but also co-resistant to fluoroquinolones and aminoglycosides (12, 13). Increasing resistance to existing antimicrobial classes underscores the importance of discovering and developing novel agents.

Bacterial DNA replication is dependent on DNA gyrase for DNA supercoiling and topoisomerase IV for chromosomal segregation that is encoded by *gyrA/gyrB* and *parC/parE* (*grlA/grlB* in *Staphylococcus* spp.), respectively (14–17). Fluoroquinolones inhibit gyrase and topoisomerase IV (16). Extensive use of fluoroquinolones due to their broad spectrum of activity against Gram-positive and Gram-negative organisms has led to widespread resistance development to agents within this antimicrobial class (18, 19). Resistance to fluoroquinolones occurs through mutations in the quinolone-resistance determining region (20–23), decreased fluoroquinolone uptake through altered bacterial membrane permeability, efflux pump overexpression (21, 24, 25), and through plasmid-mediated transfer of resistance (26). High rates of spontaneous resistance development have been observed for ciprofloxacin (27–29) and levofloxacin to a lesser extent (28).

To circumvent bacterial resistance mechanisms that have evolved after decades of intense use of fluoroquinolones, efforts have been made to design novel antimicrobials targeting DNA gyrase and topoisomerase IV. Gepotidacin is a novel, bactericidal, first-in-class triazaacenaphthylene antimicrobial that inhibits type II topoisomerases involved in bacterial DNA replication by a distinct binding site, unique mechanism of action and for most pathogens provides well-balanced inhibition of DNA gyrase and topoisomerase IV (30–34). Against target uropathogens, numerous studies have shown that gepotidacin is active *in vitro* (35, 36), *in vivo* (37), and Phase III clinical trials NCT04020341 (EAGLE-2) and NCT04187144 (EAGLE-3) showed gepotidacin to be non-inferior and superior, respectively, to nitrofurantoin for the treatment of patients with uncomplicated urinary tract infection (uUTI) (38). Based on the results of EAGLE-2 and EAGLE-3, gepotidacin was approved in March 2025 by the U.S. Food and Drug Administration (FDA) for the treatment of female adult and pediatric patients 12 years of age and older weighing at least 40 kg with uncomplicated urinary tract infections (uUTIs) caused by the following susceptible microorganisms: *Escherichia coli*, *Klebsiella pneumoniae*, *Citrobacter freundii* complex, *Staphylococcus saprophyticus,* and *Enterococcus faecalis* (39). Gepotidacin was further approved in August 2025 by the Medicines and Healthcare products Regulatory Agency (MHRA) for the treatment of uncomplicated urinary tract infections (40). It has also been developed for gonorrhea; recently, a Phase 3 clinical trial for the treatment of uncomplicated urogenital gonorrhea (NCT04010539 [EAGLE-1]) was completed, and FDA approval was obtained for uncomplicated urogenital gonorrhea in adult and pediatric patients 12 years of age and older (41).

*E. coli,* a pathogen responsible for 75% of uUTIs (42), has previously exhibited low frequency of resistance (FoR) to gepotidacin and mutant resistance mechanisms identified were drug efflux resulting in two- to fourfold higher MIC (43). Additionally, data presented by GSK at ICAAC 2013 showed FoR ranged from <8 × 10^−10^ to 2 × 10^−7^ for *S. aureus* and <9 × 10^−10^ to <7 × 10^−9^ for *E. coli*, *Streptococcus pneumoniae*, *Streptococcus pyogenes*, *Haemophilus influenzae,* and *Moraxella catarrhalis*. The purpose of this study was to evaluate the potential of gepotidacin to select for spontaneous mutations in other Gram-negative and Gram-positive uropathogens and subsequently attempt to characterize any resistance mechanisms.

## MATERIALS AND METHODS

### Organisms tested

A total of 24 clinical isolates, collected in 2019, were evaluated in this study and included three isolates each of *Citrobacter freundii*, *Enterobacter cloacae* species complex (sc) (including one *Enterobacter hormaechei* isolate)*, Klebsiella aerogenes*, *Klebsiella pneumoniae, Proteus mirabilis, Providencia rettgeri, Enterococcus faecalis,* and *Staphylococcus saprophyticus*. Organisms were selected to represent a variety of clinically relevant phenotypes including wild-type, ESBL-producing, carbapenem-resistant, and fluoroquinolone-resistant isolates found in urinary tract infections.

### Susceptibility testing and antimicrobial agents

For frequency of resistance testing and mutant colony selection, baseline agar dilution testing was performed in triplicate on Mueller-Hinton or MacConkey (*P. mirabilis* only) agar for gepotidacin and levofloxacin. FDA and CLSI interpretive criteria were applied according to current guidelines (44, 45). For determining mutant phenotype confirmation and testing for cross-resistance, baseline MICs of gepotidacin and comparator agents against selected isolates were determined using reference *in vitro* broth microdilution (BMD) testing methods, as described by Clinical and Laboratory Standards Institute (CLSI) M07 (46). BMD was performed in triplicate in cation-adjusted Mueller-Hinton broth (CAMHB) for gepotidacin, levofloxacin, azithromycin, ceftazidime, nitrofurantoin, tetracycline, and trimethoprim-sulfamethoxazole. Aztreonam and meropenem were only tested against Gram-negative organisms while linezolid and vancomycin were only tested against the Gram-positive organisms.

Quality control (QC) strains tested concomitantly with the clinical isolates included *E. coli* ATCC 25,922, *Pseudomonas aeruginosa* ATCC 27,853, and *S. aureus* ATCC 29,213. Acceptable MIC ranges for the agents tested against the QC strains were published by CLSI (44).

### Frequency of resistance

Colonies were selected from 18- to 24-h blood agar plates and suspended in CAMHB. These suspensions were adjusted to create a turbidity equivalent to a 4 McFarland standard to achieve an inoculum concentration of 1–2 × 10^9^ CFU/mL confirmed by colony counts. Then, 4× and 10× MIC gepotidacin and levofloxacin plates were inoculated with 0.1 and 1 mL from the bacterial suspension. Agar plates were incubated at 35°C in ambient conditions, and colonies were counted after 24–48 h of incubation. Plates with no drug were also inoculated to determine counts of viable bacteria and confirm purity. The FoR was calculated by dividing the number of colonies growing on the 4× and 10× agar plates by the initial inoculum concentration. Strains later confirmed to have a gepotidacin MIC value of <4-fold the baseline MIC value were excluded from the colony counts when determining resistance frequency.

### Confirmation of resistance phenotype

A selection of colonies growing on the 4× gepotidacin agar plates was subjected to confirmatory BMD MIC susceptibility testing. The selected strains were passaged twice on drug-free agar plates, identifications confirmed by matrix-assisted laser desorption ionization time of flight (MALDI ToF) and underwent additional BMD testing concurrently with parental strains to determine MIC values for gepotidacin and comparator agents. Colonies were defined as gepotidacin-resistant if BMD MIC values were ≥4-fold higher than the parent strain. A colony was determined to be cross-resistant if a ≥4-fold increase in BMD MIC was observed for a comparator agent. Mutants selected by levofloxacin were not tested for cross-resistance to gepotidacin or any comparator agents.

### Genetic characterization of resistant mutants

Select colonies demonstrating reduced gepotidacin susceptibility underwent whole genome sequencing and comparative genomics to identify potential mechanisms of resistance. Total genomic DNA was extracted and purified using the KingFisher Cell and Tissue DNA kit or the MagMAX DNA Multi-sample Ultra 2.0 kit (Thermo Scientific, Waltham, Massachusetts, USA) in a robotic KingFisher Flex Magnetic Particle Processor (Thermo Scientific) workstation. Total genomic DNA was used as input material for library construction. DNA libraries were prepared using the Nextera XT or the Illumina DNA Prep library construction and index kits (Illumina, San Diego, California, USA) and sequenced on a MiSeq Sequencer (Illumina) using a MiSeq Reagent Kit version 2 (500 cycles) or version 4 (600 cycles) or NextSeq 1000 Sequencer using NextSeq1000/2000 P2 Reagents (300 cycles). FASTQ format sequencing files for each sample set were assembled independently using *de novo* assembler SPAdes 3.13.0 (47). In-house software was applied to assembled sequences to align against target genes. All target gene sequences were considered wild-type if displaying 100.0% homology with the respective parent reference sequence. Differences showing <100.0% homology were annotated.

Resistant mutants and their respective parental isolates were sequenced using a combination of short- and long-read sequencing methods. Short-read Illumina data were produced from total genomic DNA using the above protocol. High-molecular-weight DNA was obtained using the Qiagen Genomic Tip 100/G (Qiagen, Germantown, Maryland, USA) according to manufacturer’s instructions. Sequencing library preparation was carried out with Nanopore Rapid Barcode (SQK-RBK004 or SQK-RBK-114.24) according to the manufacturer’s protocol. Sequencing was performed using either a FLO-MIN106 or FLO-MIN114 flow cell on the MinION sequencer controlled by the MinKNOW software (version 22.03.4; Oxford Nanopore Technologies Ltd., Oxford, UK).

For single nucleotide polymorphism (SNP) analysis, short- and long-reads were assembled using Unicycler version 0.4.8-beta (48). SNPs were called using MAUVE (49) version 2.4.0. All SNPs determined by MAUVE were confirmed by mapping quality trimmed reads independently to the baseline assembly. Reads were mapped using BWA version 0.7.12-r1039 (50). Insertion/deletion (INDEL) sites were realigned using IndelRealigner from the GATK toolbox version 3.8-1-gf15c13ef (51). High confidence variants were called by samtools version 1.8 and filtered by bcftools version 1.8 (52). Filtering criteria for the variant call format (VCF) file was a minimum read depth of 4 (≥2 reads per strand), >30 map quality, >50 average base quality, no significant strand bias, and >75% of mutation within reads to support the presence of any given alteration. Repeat regions of >50 bp were removed from VCF using MUMmer version 3.0 (53). Baseline assembly was annotated using Prokka version 1.14.0 (54) and SNPs were annotated using Snpeff 4.3t (55). Short reads were subjected to quality trimming using a sliding window threshold of Q18. The reference sequence was built using Unicycler’s default parameters. SNP quality metrics were then applied (56). Additional analysis was performed by nucDiff (https://omictools.com/nucdiff-tool) to capture all INDELs and uncovered regions. 

## RESULTS

### Low frequency of spontaneous resistance in Gram-negative and Gram-positive isolates exposed to 4× and 10× gepotidacin

The FoR to 4× gepotidacin ranged from 2.7 × 10^−8^ to <5.0 × 10^−9^ for *C. freundii*, 6.1 × 10^−8^ to <2.5 × 10^−10^ for *E. cloacae* sc, 2.7 × 10^−8^ to <6.6 × 10^−10^ for *K. pneumoniae*, 7.1 × 10^−10^ to <5.4 × 10^−10^ for *P. mirabilis*, 7.8 × 10^−9^ to 3.5 × 10^−9^ for *P. rettgeri*, and 1.7 × 10^−8^ to <3.2 × 10^−10^ for *E. faecalis* (Table 1). No resistant colonies were recovered for *K. aerogenes* (FoR: <4.7 × 10^−10^ to <3.7 × 10^−10^ or *S. saprophyticus* (FoR: <9.2 × 10^−10^ to <5.9 × 10^−10^) (Table 1).

**TABLE 1:** Frequency of resistance to gepotidacin and levofloxacin^*b*^

		Modal MIC (mg/mL)		Frequency of resistance rate (Resistant colonies) with:			
				GEP		LEV	
Organism	Phenotype (Genotype)	GEP	LEV	4× MIC	10× MIC	4× MIC	10× MIC
*Citrobacter freundii*							
1091286	WT	1	0.03	2.7 × 10^-8^ (17 ^*a*^)	<8.3 × 10^-10^ (0)	<9.2 × 10^-9^ (0)	<8.3 × 10^-10^ (0)
1116313	FQ-R (GyrA T83I)	8	8	5.4 × 10^-9^ (1)	<5.4 × 10^-9^ (0)	<4.9 × 10^-10^ (0)	<4.9 × 10^-10^ (0)
1130512	ESBL (CMY-180)	1	0.06	<5.0 × 10^-9^ (0)	<5.0 × 10^-9^ (0)	<4.5 × 10^-10^ (0)	<4.5 × 10^-10^ (0)
*Enterobacter cloacae* sc							
1092886	WT	4	0.06	<6.5 × 10^-10^ (0)	<6.5 × 10^-10^ (0)	<6.5 × 10^-10^ (0)	<6.5 × 10^-10^ (0)
1127328	FQ-R (GyrA S83I, QnrE3)	8	2	<2.5 × 10^-10^ (0)	<2.5 × 10^-10^ (0)	<2.5 × 10^-10^ (0)	<2.5 × 10^-10^ (0)
*Enterobacter hormaechei*							
1092279	ESBL (ACT-70)	2	0.06	6.1 × 10^-8^ (63 ^*a*^)	<5.8 × 10^-10^ (0)	5.8 × 10^-10^ (1)	<5.8 × 10^-10^ (0)
*Klebsiella aerogenes*							
1089299	ESBL (AmpC)	2	0.12	<4.7 × 10^-10^ (0)	<4.7 × 10^-10^ (0)	<4.7 × 10^-10^ (0)	<4.7 × 10^-10^ (0)
1089847	WT	2	0.06	<3.7 × 10^-10^ (0)	<3.7 × 10^-10^ (0)	5.9 × 10^-9^ (11)	<3.7 × 10^−10^ (0)
1092937	FQ-R (GyrA T83I)	4	4	<4.0 × 10^-10^ (0)	<4.0 × 10^-10^ (0)	4.0 × 10^-10^ (1)	<4.0 × 10^-10^ (0)
*Klebsiella pneumoniae*							
1098581	WT	4	0.12	6.3 × 10^-9^ (1)	<5.7 × 10^-10^ (0)	1.4 × 10^-8^ (25)	1.4 × 10^-8^ (25)
1124085	FQ-R (GyrA S83F)	16	8	2.7 × 10^-8^ (5)	<5.5 × 10^-9^ (0)	<5.0 × 10^-10^ (0)	<5.0 × 10^-10^ (0)
1130299	ESBL (CTX-M-15)	4	0.06	<6.6 × 10^-10^ (0)	<6.6 × 10^-10^ (0)	2.8 × 10^-8^ (42)	<6.6 × 10^-10^ (0)
*Proteus mirabilis*							
1091952	WT	16	0.25	7.1 × 10^-10^ (1)	<7.1 × 10^-10^ (0)	<7.1 × 10^-10^ (0)	<7.1 × 10^-10^ (0)
1106732	ESBL (CTX-M-15)	4	2	<6.4 × 10^-10^ (0)	<6.4 × 10^-10^ (0)	<6.4 × 10^-10^ (0)	<6.4 × 10^-10^ (0)
1117109	CRE (KPC-2)	64	64	<5.4 × 10^-10^ (0)	<5.4 × 10^-10^ (0)	<5.4 × 10^-10^ (0)	<5.4 × 10^-10^ (0)
*Providencia rettgeri*							
1089529	WT	4	0.12	5.4 × 10^-9^ (10)	<5.4 × 10^-10^ (0)	<5.4 × 10^-10^ (0)	<5.4 × 10^-10^ (0)
1090192	ESBL (PER-1), FQ-R (GyrA S83I, ParC S84I, QnrD1)	4	16	7.8 × 10^-9^ (17 ^*a*^)	<4.6 × 10^-10^ (0)	<4.6 × 10^-10^ (0)	<4.6 × 10^-10^ (0)
1118004	FQ-R (GyrA S83I, ParC S84I)	4	8	3.5 × 10^-9^ (8)	<4.3 × 10^-10^ (0)	5.2 × 10^-9^ (12)	<4.3 × 10^-10^ (0)
*Enterococcus faecalis*							
1097863	FQ-R (GyrA S84R, GrlA S82I)	0.5	32	<3.2 × 10^-10^ (0)	<3.2 × 10^-10^ (0)	<3.2 × 10^-10^ (0)	<3.2 × 10^-10^ (0)
1103850	WT	0.25	0.5	1.7 × 10^-8^ (2)	<8.3 × 10^-9^ (0)	<8.3 × 10^-9^ (0)	<7.6 × 10^-10^ (0)
1111210	WT	0.5	0.5	<7.0 × 10^-10^ (0)	<7.0 × 10^-10^ (0)	<7.0 × 10^-10^ (0)	<7.0 × 10^-10^ (0)
*Staphylococcus saprophyticus*							
1106006	WT	0.06	0.5	<5.9 × 10^-10^ (0)	<5.9 × 10^-10^ (0)	<5.9 × 10^-10^ (0)	<5.9 × 10^-10^ (0)
1113726	WT	0.06	0.5	<9.2 × 10^-10^ (0)	<9.2 × 10^-10^ (0)	<9.2 × 10^-10^ (0)	<9.2 × 10^-10^ (0)
1129086	WT	0.06	0.5	<8.0 × 10^-10^ (0)	<8.0 × 10^-10^ (0)	<8.0 × 10^-10^ (0)	<8.0 × 10^-10^ (0)

^*a*^ Stable, reduced susceptibility confirmed in 100% of select colonies tested; 10 out of 10 *C. freundii*colonies, 10 out 10 *E. cloacae *sc colonies, and 10 out 10 *P. rettgeri *colonies.

^*b*^ MIC, minimum inhibitory concentration; sc, species complex; CFU, colony forming units; GEP, gepotidacin; LEV, levofloxacin; CRE, carbapenem-resistant Enterobacterales; ESBL, extended-spectrum beta-lactamase; FQ-R, fluoroquinolone-resistant; WT, wild-type.

Mutants selected at 4× gepotidacin were observed from 2/3 *C. freundii*, 1/3 *E. cloacae* sc (*Enterobacter hormaechei*), 2/3 *K. pneumoniae*, 1/3 *P. mirabilis,* 3/3 *P. rettgeri*, and 1/3 *E. faecalis* isolates. Confirmatory MIC testing on these mutants from levofloxacin-susceptible parent strains revealed phenotypic cross-resistance to levofloxacin in 40% (4/10) of *E. hormaechei* mutants, 21.4% (3/14) of *K. pneumoniae* mutants, 100% (10/10) from *P. rettgeri* mutants, and in the single *P. mirabilis* mutant (Table S1). The four levofloxacin-resistant *E. hormaechei* mutants were also phenotypically cross-resistant to tetracycline and azithromycin. All but one (20/21; 95.2%) mutant from the two levofloxacin-resistant *P. rettgeri* parent isolates exhibited levofloxacin MICs that were ≥4-fold greater than respective parent MICs; Table S1). Cross-resistance to other antimicrobial agents was not observed in *Citrobacter freundii* or *E. faecalis* mutants. Target-based cross-resistance was not observed within a subset of *E. faecalis* mutants analyzed for gyrA/B and parC/E mutations (Table S1).

No resistant colonies were recovered on the 10× gepotidacin plates (<5.4 × 10^−9^ to <8.3 × 10^−10^ [*C. freundii*], <6.5 × 10^−10^ to <2.5 × 10^−10^ [*E. cloacae* sc], <4.7 × 10^−10^ to <3.7 × 10^−10^ [*K. aerogenes*], <5.5 × 10^−9^ to <5.7 × 10^−10^ [*K. pneumoniae*], <7.1 × 10^−10^ to <5.4 × 10^−10^ [*P. mirabilis*], <5.4 × 10^−10^ to <4.3 × 10^−10^ [*P. rettgeri*], <8.3 × 10^−9^ to <3.2 × 10^−10^ [*E. faecalis*], and <9.2 × 10^−10^ to <5.9 × 10^−10^ [*S. saprophyticus*] [Table 1]).

For to 4× levofloxacin ranged from <9.2 × 10^−9^ to <4.5 × 10^−10^ for *C. freundii*, <6.5 × 10^−10^ to <2.5 × 10^−10^ for *E. cloacae* sc, 5.9 × 10^−9^ to 4.0 × 10^−10^ for *K. aerogenes*, 2.8 × 10^−8^ to <5.0 × 10^−10^ for *K. pneumoniae*, <7.1 × 10^−10^ to <5.4 × 10^−10^ for *P. mirabilis*, 5.2 × 10^−9^ to <4.6 × 10^−10^ for *P. rettgeri*, <8.3 × 10^−9^ to <3.2 × 10^−10^ for *E. faecalis*, and <9.2 × 10^−10^ to <5.9 × 10^−10^ for *S. saprophyticus* (Table 1). At 10× levofloxacin, resistant colonies were recovered from only one *K. pneumoniae* isolate (FoR: 1.4 × 10^−8^). FoR to 10× levofloxacin for the remaining organisms ranged from <9.2 × 10^−10^ to <2.5 × 10^−10^.

### Reduced susceptibility to gepotidacin is linked to alterations in efflux pump-associated regulatory proteins

To define potential mechanisms contributing to reduced gepotidacin susceptibility of mutants selected on 4× gepotidacin plates, whole-genome sequencing and comparative genomics were used to identify SNPs and small insertions/deletions. For this analysis, 1–2 mutants of each species were sequenced to determine chromosomal variations from their respective parental strain.

*C. freundii* isolate #1091286 (gepotidacin MIC, 1 mg/L) produced mutant isolate #1091286-162 which displayed a 16-fold decrease in gepotidacin susceptibility (MIC, 16 mg/L). Susceptibility to levofloxacin was not dramatically altered in the mutant (MIC, 0.03 mg/L vs 0.015 mg/L for parent and mutant, respectively; Table 2). Analysis of accumulated genetic alterations revealed the presence of a single cytosine deletion (ΔC483), producing a frameshift mutation and early termination codon (L162fs_L168X) in a gene encoding a putative TetR-family transcription factor with 94.8% identity to the major facilitator superfamily (MFS) efflux pump regulator SmvR in *C. freundii* ATCC 8090. Additional alterations were noted in the F_0_F_1_ ATP synthetase α-subunit, AtpA (R101P), 46-bp upstream of a putative hemolysin-encoding gene (ΔT2181646), and in the intergenic region between *oxyR* and a putative glycosyl hydrolase-encoding gene (G4649535A) (Table 2).

**TABLE 2:** Summary of antimicrobial susceptibility and accumulated mutations in select sequenced mutant isolates exhibiting reduced gepotidacin susceptibility^*d*^

Organism-ID^*a*^	MIC mg/L (fold change)^*b*^		Mutation analysis^*c*^
	GEP	LEV	
*C. freundii* 1091286 (P)	1	0.03	
1091286-162	16 (16)	0.015 (0.5)	SmvR L162fs_L168X, MFS efflux repressor; AtpA R101P, ATP synthesis; intergenic oxyR (+362) -glycosyl transferase (+121); upstream putative hemolysin (+46); adenylosuccinate lyase – PurB A280E
*E. hormaechei* 1092279 (P)	4	0.03	
1092279-137	***64*** (16)	0.5 (16)	Intergenic ΔT TetR family repressor (+126) RamR-RomA family hydrolase (+66)
1092279-143	***32*** (8)	0.03 (-)	SmvR ΔT36-A41, MFS efflux repressor
*K. pneumoniae* 1124085 (P)	***32***	8	
1124085-210	***256*** (8)	64 (8)	OqxR G63_S64insSIHLGRNG, RND efflux regulator; RpoC R431P, RNA polymerase subunit
1124085-213	***128*** (4)	16 (2)	OqxR G63_S64insSIHLGRNG, RND efflux regulator; RpoC K334Q, RNA polymerase subunit
*P. mirabilis* 1091952 (P)	16	0.06	
1091952-224	***128*** (8)	0.5 (8)	SoxR R20L, oxidative stress response transcriptional activator; predicted MFS transporter I158F, TetR/AcrR family transcriptional regulator; upstream hypothetical protein T3696876A; between hypothetical and uncharacterized protein T2113293C
*P. rettgeri* 1089529 (*P*)	4	0.12	
1089529-174	128 (32)	1 (8)	SoxR R33C, oxidative stress response transcriptional activator; downstream intergenic suhB-Zn alcohol dehydrogenase; response regulator UvrY; LysR family transcriptional regulator, N121Il; LysR family transcriptional regulator E118V; LysR family transcriptional regulator D117E; Glycine cleavage system T protein and Ubiquinone biosynthesis hydroxylase, UbiH; type II secretion system F family protein; downstream GyrB; Homoserine trans-succinylase [Amino acid transport and metabolism]; Multidrug-resistant protein MdtN; Formate dehydrogenase-O major subunit
*E. faecalis* 1103850 (P)	0.25	0.5	
1103850-135	4 (16)	0.5 (-)	GyrB D437V, DNA gyrase subunit B
1103850-136	2 (8)	0.5 (-)	GyrA A34V, DNA gyrase subunit A

^*a*^ Parental isolates are denoted with a (P); mutant isolates are right-indented.

^*b*^ Isolates demonstrating identical MIC values relative to the parental strain are denoted with (-).

^*c*^ Positions of intergenic alterations are given relative to the predicted translational start sites or stop codons of the flanking gene.

^*d*^ MIC, minimum inhibitory concentration; GEP, gepotidacin; LEV, levofloxacin; fs, frameshift; ins, insertion. Bold and italic values denote mutant MIC values (mg/L) that are nonsusceptible by US FDA or CLSI interpretative criteria (45, 57).

Parental isolate *E. hormaechei* #1092279 (gepotidacin MIC, 4 mg/L) produced two mutant isolates, #1092279-137 and #1092279-143, with gepotidacin MIC values of 64 and 32 mg/L, respectively. The activity of levofloxacin against #1092279-143 was similar to that against the parent strain, whereas #1092279-137 displayed a 16-fold decrease in levofloxacin susceptibility (MIC, 0.5 mg/L). Mutant #1092279-143 displayed an 18-bp deletion encompassing residues T36-A41 of SmvR. Mutant #1092279-137 acquired an alteration (ΔT1196444) in the intergenic space between the TetR/AcrR-family transcriptional regulator RamR (+126 from predicted translational start site) and a RomA metallohydrolase encoding gene (+66; Table 2).

Two mutants from *K. pneumoniae* strain #1124085 (gepotidacin MIC, 32 mg/L), #1124085-210 (256 mg/L) and #1124085-213 (128 mg/L), were characterized for their four- to eightfold increases in gepotidacin MIC values. Each isolate possessed an eight-residue insertion in the *oqxAB* efflux pump repressor OqxR (G63_S64insSIHLGRNG). Furthermore, each isolate possessed a unique alteration in the RNA polymerase subunit-encoding gene *rpoC* producing R431P (#1124085-210) or K334Q (#1124085-213) variants and a shared alteration (G4739328T) in the intergenic region between two convergently-oriented hypothetical genes. The different alterations in RpoC may account for the fourfold difference in levofloxacin MIC values between mutant isolates (Table 2).

Five alterations were identified in *P. mirabilis* #1091952-224 (gepotidacin MIC, 128 mg/L), the mutant isolate of *P. mirabilis* parental strain #1091952 (MIC, 16 mg/L). An R20L substitution was identified in the redox-sensitive transcriptional activator SoxR, which activates transcription of SoxS, ultimately leading to upregulation of numerous efflux pump systems, including the multi-drug targeting *acrAB-tolC* system. Upregulation of *acrAB* may have also contributed to the eightfold decrease in levofloxacin susceptibility in #1091952-224. A second I158F mutation was identified in a putative MFS transporter (Table 2).

An alteration in SoxR (R33C) was also identified in *P. rettgeri* mutant isolate #1089529-174 (gepotidacin MIC, 128 mg/L; parental strain #1089529 gepotidacin MIC, 4 mg/L) in addition to an alteration in the intergenic region between the convergently transcribed *suhB* and a gene encoding a putative Zn-binding alcohol dehydrogenase. The R33C alteration in *soxR* may have exerted a similar effect on levofloxacin susceptibility in #1089529-174 as was noted for the *P. mirabilis* mutant #1091952-224 with an eightfold MIC increase compared with the parental strain (Table 2).

Finally, regarding Gram-positives, two mutants were recovered from *E. faecalis* #1103850 (#1103850-135 [MIC, 4 mg/L] and #1103850-136 [MIC, 2 mg/L]; parental strain gepotidacin MIC, 0.25 mg/L) from 4× MIC gepotidacin plates. Both mutants #1103850-135 and #1103850-136 each had an alteration in DNA gyrase, #1103850-135 had a GyrB D437V substitution and #1103850-136 had a GyrA A34V substitution (Table 2). While these mutations led to an increase in gepotidacin MIC values, both isolates would still be interpreted as susceptible to gepotidacin based on FDA or EUCAST breakpoints (Table S1). No increases in levofloxacin MICs were observed for either *E. faecalis* mutant.

## DISCUSSION

The ever-evolving threat of antimicrobial resistance necessitates the continued development of antimicrobials with novel targets and/or mechanisms of action. Gepotidacin is a novel, first-in-class, triazaacenaphthylene antibiotic that inhibits bacterial DNA replication by inhibition of DNA gyrase and topoisomerase IV via a distinct binding site (when compared to fluoroquinolones), a unique mechanism of action and for most pathogens, well-balanced inhibition of both Type II topoisomerases (30–33). This provides *in vitro* activity against most target pathogens, including those resistant to standard of care antibiotics (35, 36). Multiple older classes of antimicrobials, including the quinolones, similarly target bacterial DNA replication machinery, but use of these agents over time has resulted in the spread of transferable resistance mechanisms and the allelic fixation of target site chromosome-based mutations in common UTI-associated clones (58). Although gepotidacin has demonstrated activity against numerous species (36, 59) and phenotypic drug-resistant subsets, including quinolone-resistant isolates (36, 60), mechanisms of resistance to this agent have not been fully explored. Here, we determined the FoR to gepotidacin in diverse clinical bacterial isolates following exposure to gepotidacin at 4× and 10× MIC. Furthermore, when gepotidacin selection resulted in the generation of isolates demonstrating stably reduced susceptibility, we used whole genome sequencing and comparative genomics to identify potential mechanisms of resistance.

Among Gram-negative isolates exposed to gepotidacin at 4× MIC, frequencies of spontaneous resistance ranged from 6.1 × 10^−8^ to <2.5 × 10^−10^; FoR to levofloxacin was comparable, ranging from 2.8 × 10^−8^ to <2.5 × 10^−10^. At least one isolate from each species exposed to gepotidacin at 4× MIC, except *K. aerogenes*, produced mutant isolates with reduced gepotidacin susceptibility, while gepotidacin at 10× MIC prevented the emergence of resistant colonies across all species and for all but *K. pneumoniae* when exposed to levofloxacin at 10× MIC. Interestingly, some isolates that were selected at 4× MIC were shown to confer a 16- to 32-fold increase in gepotidacin MIC. It is not clear whether the testing methods between these two results (agar vs broth MIC) or differences in selective pressure/inoculum effects (single cell growing to a visible colony on a plate vs stable cell population at 5 × 10^5^ CFU/mL) are responsible for this observation. It should be noted that for all species other than *P. rettgeri*, 88% of the characterized mutants showed ≤8-fold increase in MIC and would not expect to be recovered on 10× agar plates. Reasons for differences observed with *P. rettgeri* are unknown, although this species is often cited for its genomic plasticity in resistance development (61).

Following FoR selection, one or two mutants derived from six different Enterobacterales species, demonstrating elevated and stable gepotidacin MIC values, were characterized for potential mechanisms of resistance. No target-based mutations within GyrA/B and ParC/E were found among the Enterobacterales isolates analyzed. Overall, mutations were identified in or near transcriptional regulators known to affect the activities of various multi-drug targeting efflux pump systems. Specifically, *K. pneumoniae* isolates acquired mutations in the *oqxAB* RND efflux pump repressor *oqxR, P. mirabilis* and *P. rettgeri* isolates displayed mutations in the activator *soxR,* the *E. hormaechei* isolate and one *C. freundii* isolate had mutations in the *smvA* MFS efflux pump repressor *smvR,* and the *E. hormaechei* isolate had an alteration upstream of the multi-efflux pump and porin repressor *ramR*. These results suggest that multiple efflux pump systems and their associated regulatory networks may influence extrusion of gepotidacin from the cell, and that these mechanisms vary across Gram-negative species.

OqxR is a GntR-family repressor of the *oqxAB* efflux pump system in *K. pneumoniae* (62). OqxAB, an RND family multi-drug efflux pump system chromosomally located in *K. pneumoniae* (63) and later found in conjugative plasmids in other Enterobacterales isolates (64), targets multiple antimicrobial classes for cellular extrusion (65–67). Inactivation of *oqxR* promotes *oqxAB* derepression and concomitant increases in MIC values for fluoroquinolones (68), nitrofurantoin (69), and chloramphenicol among other antimicrobials (70). Mutations, including missense point mutations, insertion of mobile genetic elements, and large duplications/deletions across the entire *oqxR* coding sequence, have been associated with reduced susceptibility to those agents expelled by OqxAB and have been identified in *in vitro* studies (69, 71) and in clinical isolates (70). Mutant isolates #1124085-210 and #1124085-213, bearing identical 8-amino acid insertions (G63_S64insSIHLGRNG) in *oqxR*, demonstrated four- to eightfold reductions in gepotidacin susceptibility. This mutation was previously shown to increase *oqxAB* expression in *K. pneumoniae* following *in vitro* selection for reduced susceptibility to fluoroquinolones (71). However, only #1124085-210 showed a ≥4-fold decrease in susceptibility to levofloxacin. Taken together, these results lend support that *oqxR-*inactivating mutations can be selected for using a variety of agents, therefore decreasing susceptibility to those agents.

The *P. mirabilis* and *P. rettgeri* isolates subjected to FoR accrued unique mutations in a gene encoding a putative MerR family transcriptional regulator with ~60% identity to the *E. coli* redox-sensitive transcriptional activator SoxR. In *E. coli,* oxidation of the C-terminal Fe-S cluster in SoxR homodimers elicits conformational changes promoting transcriptional activation of the divergently transcribed *soxS,* an activator of the multi-drug efflux pump system *acrAB* (72). Numerous studies of clinical isolates and isolates evolved *in vitro* following antimicrobial exposure have outlined the role of *soxR* mutations in driving SoxS-mediated reduced susceptibility to fluoroquinolones through *acrAB* overexpression, among other mechanisms (73–75). Although the mutations identified in *P. mirabilis* #1091952-224 (SoxR^R20L^; gepotidacin MIC, 128 mg/L) and *P. rettgeri* #1089529-174 (SoxR^R33C^; MIC, 128 mg/L) are located in the N-terminal DNA-binding domain and would presumably affect the ability of SoxR to activate transcription, R20C and R20H mutations have been shown to produce constitutively active SoxR variants, promoting elevated *soxS* expression and antimicrobial resistance (76). The R20 residue, and potentially the *P. rettgeri* R33 residue, which corresponds to R20 in *P. mirabilis* SoxR due to a 17-residue N-terminal extension of the *P. rettgeri* coding sequence, appears to manifest in resistance profiles similar to those of *E. coli* bearing constitutively active SoxR variants*,* as MIC values for levofloxacin, a substrate of AcrAB, were elevated 8-fold in each isolate with *soxR* mutations. Interestingly, the genes encoding the putative SoxR regulators identified in *P. mirabilis* and *P. rettgeri* lack the canonical *soxR-soxS* genomic organization, with no proximal *soxS* gene identified in either strain. Rather, immediately upstream in both isolates lay three divergently organized genes predicted to encode a DoxX domain-containing transmembrane protein, an RND family permease, and an RND family adaptor protein. Therefore, the SoxR-like proteins identified here may constitute a redox-sensitive transcriptional regulator controlling an uncharacterized efflux pump system targeting gepotidacin for removal from the cell. Further investigations of the activities and distribution of the regulator and pump system identified in these two species may provide insight into the unique nature of reduced gepotidacin susceptibility in members of the *Morganellaceae* compared with the family *Enterobacteriaceae*.

Alterations in the TetR family regulator SmvR were identified in one *E. hormaechei* #1092279-143 (SmvR^ΔT36-A41^; gepotidacin MIC, 32 mg/L) and *C. freundii* #1091286-162 (SmvR^L162fs_L168STOP^; MIC, 16 mg/L). SmvR represses the expression of the MFS family efflux pump *smvA,* which functions to remove cationic biocides (e.g., chlorhexidine) and reactive oxygen species-producing molecules (e.g., methyl viologen) from the cytoplasm (77). Expression of *smvA* increases in the presence of target substrates or through mutation of *smvR* (78, 79). *P. mirabilis* (80)*, C. freundii* (79)*, K. pneumoniae* (81)*,* and *E. cloacae* (82) isolates obtained through *in vitro* exposure studies and from clinical sources have further demonstrated the adaptive nature of the *smvR/smvA* system following exposure to target substates. Relatedly, isolate *E. hormaechei* #1092279-137 (gepotidacin MIC, 64 mg/L) bore a single alteration in the 191-bp intergenic space separating genes encoding the TetR/AcrR family repressor RamR and a RomA family hydrolase. Reduced susceptibility to numerous antimicrobial classes has been demonstrated previously in clinical and *in vitro-*generated *E. cloacae* sc isolates lacking RamR-mediated repression of efflux pump regulators (*ramA*), pumps (*acrAB-tolC*), and porins (*ompF*)*,* suggesting the alteration identified in #1092279-137 may affect the expression of *ramR* itself (83, 84).

The Gram-positive isolates tested in this study were generally unable to produce mutants under selection with gepotidacin or levofloxacin, as only two isolates, both derived from *E. faecalis* #1,103,850, were obtained. However, those isolates, #1103850-135 (gepotidacin MIC, 4 mg/L) and #1103850-136 (MIC, 2 mg/L), represent the only target-site mutations in GyrB (D437V) and GyrA (A34V), respectively, obtained in this study. Similar to the Enterobacterales mutants characterized, no mutations were observed in ParC or ParE. Although previous studies examining the interaction of gepotidacin and related compounds with *S. aureus* GyrA/GyrB did not implicate the residues identified in this study as important for the activity of gepotidacin (30, 85), *S. aureus* N315 exposed to the investigational type II topoisomerase inhibitor NXL101 did produce a mutant bearing a D437V alteration in GyrB, resulting in an 8-fold reduction in NXL101 susceptibility (86). Further mechanistic studies could shed light on the function of these two residues in reducing susceptibility to gepotidacin. The dearth of target mutations found in other isolates tested in this study may be due to gepotidacin’s balanced targeting of gyrase and topoisomerase IV limiting the emergence of target-mediated resistance. However, while a dual targeting mechanism may reduce the development of target mutations, it doesn’t prevent the enhanced efflux response demonstrated in the study strains as seen by the preponderance of overexpressed and mutated efflux pumps.

There were some limitations to this study. While phenotypic cross-resistance to levofloxacin was observed, a limited number of mutants were selected for SNP analysis. Identification of these SNPs is more descriptive rather than mechanistic, and further genetic studies, including isogeneic strains, are needed to confirm causation of both decreased gepotidacin activity and cross-resistance. Our SNP analysis can only detect stable chromosomal mutations and would not be able to identify other mutations that could lead to higher MIC values, such as gene duplication events, increasing the gene copy number for efflux pumps. Also, further genetic analyses would be necessary to identify potential mechanisms of resistance in the remaining non-sequenced mutants. Finally, while these mutations occurred after exposure to gepotidacin at 4× MIC *in vitro*, whether these resistance mechanisms could evolve in a clinical setting, when gepotidacin concentrations in urine are often far greater than fourfold the MIC during treatment, is unknown (87, 88).

In conclusion, this study represents one of the first broad examinations of evolved resistance following exposure to gepotidacin *in vitro* on many less prevalent uropathogen species. In general, the frequency of spontaneous resistance was low in isolates exposed to 4× gepotidacin, and no evidence of spontaneous resistance was observed in isolates exposed to 10× gepotidacin. No target-based cross-resistance was observed with any comparator antimicrobial agents. Mechanisms of resistance in Gram-negative isolates coalesced around the potential contributions of overexpressed efflux pumps derived from repressor inactivation (e.g., OqxR, SmvR, and RamR) or activator dysregulation (e.g., SoxR). Mutations in regulators previously shown to control the multi-drug RND family efflux systems *oqxAB* and *acrAB* and the biocide efflux system *smvA* were found across Gram-negative isolates. Furthermore, *E. faecalis* mutants displayed target mutations in both subunits of DNA gyrase, albeit these mutants remained susceptible to gepotidacin based on FDA breakpoints and showed no changes in levofloxacin susceptibility. Importantly, this work defines a relatively small set of species-specific genes to serve as the basis for surveillance of circulating genotypes among clinical bacterial isolates against which gepotidacin may represent a viable treatment option.

## Data Availability

Raw reads have been deposited in the NCBI Sequence Read Archive under BioProject accession number PRJNA1432997. Assembled genomes have been deposited in GenBank under BioSample accessions numbers SAMN56353158 to SAMN56353172.

## SUPPLEMENTAL MATERIAL

Table S1 aac.01832-25-s0001.docx MIC values denoting activity of gepotidacin and comparators against baseline isolates and mutants that grew on gepotidacin-containing agar plates.

ASM does not own the copyrights to Supplemental Material that may be linked to, or accessed through, an article. The authors have granted ASM a non-exclusive, world-wide license to publish the Supplemental Material files. Please contact the corresponding author directly for reuse.
