# Supplementary material for: Determination of frequency of spontaneous resistance for gepotidacin and levofloxacin against a collection of gram-positive and gram-negative organisms
Source: Antimicrob Agents Chemother. 2026 Apr 30;70(6):e01832-25. doi: 10.1128/aac.01832-25 (PMC13231899; doi:10.1128/aac.01832-25)
Supplement: Table S1 — MIC values denoting activity of gepotidacin and comparators against baseline isolates and mutants that grew on gepotidacin-containing agar plates. [file aac.01832-25-s0001.docx]

**Table S1.** Activity of gepotidacin and comparators against baseline isolates and mutants that grew on gepotidacin-containing agar plates

|  |  |  | **MIC (mg/L)** | | | | | | | | | | |
| --- | --- | --- | --- | --- | --- | --- | --- | --- | --- | --- | --- | --- | --- |
| **Isolate** | **Organism** | **Exposure** | **GEP** | **LEV** | **AZI** | **CAZ** | **NIT** | **TET** | **TMP-SMX** | **ATM** | **MEM** | **LZD** | **VAN** |
| 1091286 | *C. freundii* | Baseline | 1 | 0.03 | 4 | 0.25 | 32 | 1 | ≤0.12 | 0.12 | ≤0.03 |  |  |
| 1091286-161 | *C. freundii* | 4xAgar | **8** | 0.015 | 4 | 0.25 | 16 | 1 | ≤0.12 | 0.06 | ≤0.03 |  |  |
| 1091286-162 | *C. freundii* | 4xAgar | **16** | 0.015 | 2 | 0.25 | 32 | 1 | ≤0.12 | 0.06 | ≤0.03 |  |  |
| 1091286-163 | *C. freundii* | 4xAgar | **8** | 0.015 | 4 | 0.25 | 16 | 1 | ≤0.12 | 0.06 | ≤0.03 |  |  |
| 1091286-164 | *C. freundii* | 4xAgar | **8** | 0.015 | 8 | 0.25 | 16 | 1 | ≤0.12 | 0.06 | ≤0.03 |  |  |
| 1091286-165 | *C. freundii* | 4xAgar | **8** | 0.015 | 4 | 0.25 | 16 | 1 | ≤0.12 | 0.12 | ≤0.03 |  |  |
| 1091286-166 | *C. freundii* | 4xAgar | **8** | 0.015 | 8 | 0.5 | 16 | 1 | ≤0.12 | 0.06 | ≤0.03 |  |  |
| 1091286-167 | *C. freundii* | 4xAgar | **16** | 0.03 | 8 | 0.25 | 16 | ≤0.5 | ≤0.12 | 0.12 | ≤0.03 |  |  |
| 1091286-168 | *C. freundii* | 4xAgar | **8** | 0.015 | 8 | 0.25 | 16 | 1 | ≤0.12 | 0.12 | ≤0.03 |  |  |
| 1091286-169 | *C. freundii* | 4xAgar | **8** | 0.015 | 4 | 0.25 | 16 | 1 | ≤0.12 | 0.06 | ≤0.03 |  |  |
| 1091286-170 | *C. freundii* | 4xAgar | **8** | 0.015 | 2 | 0.25 | 16 | 1 | ≤0.12 | 0.06 | ≤0.03 |  |  |
| 1116313 | *C. freundii* | Baseline | 16 | 8 | 64 | 1 | 32 | *>128* | >16 | 0.25 | ≤0.03 |  |  |
| 1116313-222 | *C. freundii* | 4xAgar | ***128*** | 8 | 32 | 1 | 32 | *8* | 1 | 0.25 | ≤0.03 |  |  |
| 1130512 | *C. freundii* | Baseline | 2 | 0.03 | 16 | *64* | 16 | 1 | ≤0.12 | *16* | ≤0.03 |  |  |
| 1130512-158 | *C. freundii* | 4xAgar | 2 | 0.06 | 16 | *>64* | 16 | 1 | ≤0.12 | *16* | ≤0.03 |  |  |
| 1092279 | *E. hormaechei* | Baseline | 4 | 0.03 | 8 | *64* | 32 | 1 | ≤0.12 | *32* | ≤0.03 |  |  |
| 1092279-137 | *E. hormaechei* | 4xAgar | ***64*** | **0.5** | **64** | *64* | *64* | ***16*** | 0.25 | *32* | 0.06 |  |  |
| 1092279-139 | *E. hormaechei* | 4xAgar | ***32*** | **0.5** | **32** | *64* | *64* | ***16*** | 0.25 | *32* | ≤0.03 |  |  |
| 1092279-141 | *E. hormaechei* | 4xAgar | ***32*** | **0.25** | **32** | *64* | *64* | ***16*** | ≤0.12 | *32* | ≤0.03 |  |  |
| 1092279-143 | *E. hormaechei* | 4xAgar | ***32*** | 0.03 | 16 | *64* | 32 | 2 | ≤0.12 | *32* | ≤0.03 |  |  |
| 1092279-146 | *E. hormaechei* | 4xAgar | **16** | **0.25** | **32** | *32* | *64* | ***16*** | ≤0.12 | *16* | 0.06 |  |  |
| 1092279-147 | *E. hormaechei* | 4xAgar | ***32*** | 0.03 | 16 | *64* | 32 | 2 | ≤0.12 | *32* | ≤0.03 |  |  |
| 1092279-148 | *E. hormaechei* | 4xAgar | ***64*** | 0.03 | 16 | *64* | 32 | 2 | ≤0.12 | *32* | ≤0.03 |  |  |
| 1092279-149 | *E. hormaechei* | 4xAgar | ***32*** | 0.06 | 16 | *64* | *64* | 2 | ≤0.12 | *32* | 0.06 |  |  |
| 1092279-150 | *E. hormaechei* | 4xAgar | ***32*** | 0.03 | 16 | *64* | 32 | 2 | ≤0.12 | *32* | ≤0.03 |  |  |
| 1092279-153 | *E. hormaechei* | 4xAgar | ***32*** | 0.03 | 16 | *>64* | *64* | 2 | ≤0.12 | *32* | ≤0.03 |  |  |
| 1098581 | *K. pneumoniae* | Baseline | 4 | 0.06 | 8 | 0.25 | 32 | 1 | ≤0.12 | 0.06 | ≤0.03 |  |  |
| 1098581-182 | *K. pneumoniae* | 4xAgar | ***32*** | 0.12 | 16 | 0.5 | *64* | 2 | 0.25 | 0.12 | ≤0.03 |  |  |
| 1124085 | *K. pneumoniae* | Baseline | 32 | 8 | 8 | 0.25 | *>128* | 4 | 2 | 0.12 | ≤0.03 |  |  |
| 1124085-210 | *K. pneumoniae* | 4xAgar | ***256*** | **64** | 4 | 0.25 | *>128* | 4 | *4* | 0.12 | 0.06 |  |  |
| 1124085-211 | *K. pneumoniae* | 4xAgar | ***256*** | **64** | 4 | 0.25 | *>128* | 4 | *4* | 0.12 | 0.06 |  |  |
| 1124085-212 | *K. pneumoniae* | 4xAgar | 64 | 8 | 8 | 0.25 | *>128* | 4 | 2 | 0.12 | ≤0.03 |  |  |
| 1124085-213 | *K. pneumoniae* | 4xAgar | ***128*** | 16 | 8 | 0.5 | *>128* | 4 | 1 | 0.12 | ≤0.03 |  |  |
| 1124085-214 | *K. pneumoniae* | 4xAgar | 64 | 8 | 8 | 0.25 | *>128* | 4 | 2 | 0.12 | ≤0.03 |  |  |
| 1124085-215 | *K. pneumoniae* | 4xAgar | ***128*** | 16 | 8 | 0.25 | *>128* | 4 | *4* | 0.12 | 0.06 |  |  |
| 1124085-216 | *K. pneumoniae* | 4xAgar | 64 | 8 | 8 | 0.25 | *>128* | 4 | 2 | 0.12 | ≤0.03 |  |  |
| 1124085-218 | *K. pneumoniae* | 4xAgar | ***128*** | **32** | 8 | 0.25 | *>128* | 4 | *4* | 0.06 | 0.06 |  |  |
| 1124085-219 | *K. pneumoniae* | 4xAgar | 64 | 8 | 8 | 0.25 | *>128* | 4 | 2 | 0.25 | ≤0.03 |  |  |
| 1091952 | *P. mirabilis* | Baseline | 16 | 0.06 | 64 | 0.06 | *128* | *32* | 2 | ≤0.03 | 0.12 |  |  |
| 1091952-224 | *P. mirabilis* | 4xAgar | ***128*** | **0.5** | 64 | 0.06 | *128* | *32* | 2 | ≤0.03 | 0.06 |  |  |
| 1089529 | *P. rettgeri* | Baseline | 4 | 0.12 | 128 | 0.06 | 32 | *64* | ≤0.12 | ≤0.03 | ≤0.03 |  |  |
| 1089529-172 | *P. rettgeri* | 4xAgar | ***64*** | **1** | 128 | 0.06 | 32 | *64* | ≤0.12 | ≤0.03 | ≤0.03 |  |  |
| 1089529-173 | *P. rettgeri* | 4xAgar | ***128*** | **1** | 128 | 0.06 | 32 | *128* | ≤0.12 | ≤0.03 | ≤0.03 |  |  |
| 1089529-174 | *P. rettgeri* | 4xAgar | ***128*** | **1** | 128 | 0.06 | 32 | *128* | 0.25 | ≤0.03 | ≤0.03 |  |  |
| 1089529-175 | *P. rettgeri* | 4xAgar | ***128*** | **1** | 128 | 0.06 | 32 | *64* | 0.25 | ≤0.03 | ≤0.03 |  |  |
| 1089529-176 | *P. rettgeri* | 4xAgar | ***128*** | **1** | 128 | 0.06 | 32 | *64* | 0.25 | ≤0.03 | ≤0.03 |  |  |
| 1089529-177 | *P. rettgeri* | 4xAgar | ***128*** | **2** | >128 | 0.06 | 32 | *64* | 0.25 | ≤0.03 | 0.06 |  |  |
| 1089529-178 | *P. rettgeri* | 4xAgar | ***128*** | **1** | 128 | 0.06 | 32 | *128* | ≤0.12 | ≤0.03 | ≤0.03 |  |  |
| 1089529-179 | *P. rettgeri* | 4xAgar | ***64*** | **1** | 128 | 0.12 | 32 | *128* | 0.25 | ≤0.03 | ≤0.03 |  |  |
| 1089529-180 | *P. rettgeri* | 4xAgar | ***64*** | **1** | 128 | 0.06 | 32 | *128* | ≤0.12 | ≤0.03 | ≤0.03 |  |  |
| 1089529-181 | *P. rettgeri* | 4xAgar | ***128*** | **1** | 128 | 0.06 | 32 | *128* | ≤0.12 | ≤0.03 | ≤0.03 |  |  |
| 1090192 | *P. rettgeri* | Baseline | 4 | 16 | 64 | *>64* | *>128* | *64* | *>16* | *16* | ≤0.03 |  |  |
| 1090192-184 | *P. rettgeri* | 4xAgar | ***64*** | **64** | 64 | *>64* | *>128* | *64* | *>16* | *16* | ≤0.03 |  |  |
| 1090192-185 | *P. rettgeri* | 4xAgar | ***64*** | **64** | 64 | *>64* | *>128* | *64* | *>16* | *16* | ≤0.03 |  |  |
| 1090192-186 | *P. rettgeri* | 4xAgar | ***64*** | **64** | 64 | *>64* | *>128* | *64* | *>16* | *16* | ≤0.03 |  |  |
| 1090192-187 | *P. rettgeri* | 4xAgar | ***64*** | **64** | 64 | *>64* | *>128* | *64* | *>16* | *16* | ≤0.03 |  |  |
| 1090192-188 | *P. rettgeri* | 4xAgar | ***64*** | **>64** | >128 | *>64* | *>128* | *128* | *>16* | *32* | ≤0.03 |  |  |
| 1090192-189 | *P. rettgeri* | 4xAgar | ***64*** | **>64** | 64 | *>64* | *>128* | *64* | *>16* | *32* | ≤0.03 |  |  |
| 1090192-190 | *P. rettgeri* | 4xAgar | ***64*** | **64** | 64 | *>64* | *>128* | *64* | *>16* | *16* | ≤0.03 |  |  |
| 1090192-191 | *P. rettgeri* | 4xAgar | ***64*** | **64** | 64 | *>64* | *>128* | *32* | *>16* | *16* | ≤0.03 |  |  |
| 1090192-192 | *P. rettgeri* | 4xAgar | ***128*** | **>64** | 64 | *>64* | *>128* | *64* | *>16* | *32* | ≤0.03 |  |  |
| 1090192-193 | *P. rettgeri* | 4xAgar | ***128*** | **>64** | 64 | *>64* | *>128* | *64* | *>16* | *16* | ≤0.03 |  |  |
| 1090192-194 | *P. rettgeri* | 4xAgar | ***128*** | **>64** | 64 | *>64* | *>128* | *64* | *>16* | *16* | ≤0.03 |  |  |
| 1090192-203 | *P. rettgeri* | 4xAgar | ***128*** | **64** | 64 | *>64* | *>128* | *64* | *>16* | *16* | ≤0.03 |  |  |
| 1118004 | *P. rettgeri* | Baseline | 4 | 8 | 64 | 0.06 | *>128* | *64* | *>16* | ≤0.03 | 0.06 |  |  |
| 1118004-195 | *P. rettgeri* | 4xAgar | 8 | 8 | **>128** | 0.06 | *>128* | *32* | *>16* | ≤0.03 | 0.06 |  |  |
| 1118004-196 | *P. rettgeri* | 4xAgar | ***128*** | **>64** | 64 | 0.12 | *>128* | *64* | *>16* | ≤0.03 | ≤0.03 |  |  |
| 1118004-197 | *P. rettgeri* | 4xAgar | ***64*** | **>64** | 64 | 0.06 | *>128* | *64* | *>16* | ≤0.03 | ≤0.03 |  |  |
| 1118004-198 | *P. rettgeri* | 4xAgar | ***128*** | **>64** | 128 | 0.06 | *>128* | *128* | *>16* | ≤0.03 | 0.06 |  |  |
| 1118004-199 | *P. rettgeri* | 4xAgar | ***64*** | **>64** | 64 | 0.06 | *>128* | *64* | *>16* | ≤0.03 | ≤0.03 |  |  |
| 1118004-200 | *P. rettgeri* | 4xAgar | ***128*** | **>64** | 64 | 0.06 | *>128* | *64* | *>16* | ≤0.03 | ≤0.03 |  |  |
| 1118004-201 | *P. rettgeri* | 4xAgar | ***128*** | **>64** | 64 | 0.06 | *>128* | *64* | *>16* | ≤0.03 | ≤0.03 |  |  |
| 1118004-202 | *P. rettgeri* | 4xAgar | ***128*** | **>64** | 64 | 0.06 | *>128* | *64* | *>16* | ≤0.03 | ≤0.03 |  |  |
| 1118004-225 | *P. rettgeri* | 4xAgar | ***64*** | **32** | 32 | 0.06 | *64* | *16* | *>16* | ≤0.03 | ≤0.03 |  |  |
| 1103850 | *E. faecalis* | Baseline | 0.25 | 0.5 | >128 | >64 | ≤8 | *32* | 0.25 |  |  | 1 | 1 |
| 1103850-135 | *E. faecalis* | 4xAgar | **4** | 0.5 | >128 | >64 | ≤8 | *32* | 0.25 |  |  | 1 | 1 |
| 1103850-136 | *E. faecalis* | 4xAgar | **2** | 0.5 | >128 | >64 | 16 | *32* | ≤0.12 |  |  | 1 | 1 |

Antimicrobial agent abbreviations: GEP, gepotidacin; LEV, levofloxacin; AZI, azithromycin; CAZ, ceftazidime; NIT, nitrofurantoin; TET, tetracycline; TMP-SMX, trimethoprim-sulfamethoxazole; ATM, aztreonam; MEM, meropenem; LZD, linezolid; VAN, vancomycin.
Bold values denote a ≥4-fold increase compared to the parent baseline MIC value. Bold and italic values denote mutant MIC values (mg/L) with a ≥4-fold increase compared to the parent baseline MIC value and were also not susceptible by US FDA or CLSI interpretative criteria [39, 84].
